# The Journal’s Premiums

**Published:** 1896-01

**Authors:** 


### THE JOURNAL'S PREMIUMS.

Subscribers will find it decidedly to their interest to take advantage of our excellent premium offers. We charge you nothing extra for a premium, as we get their cost in the pleasure of having a well-paid-up subscription list.

(1) TO EACH NEW SUBSCRIBER PAYING A YEAR'S SUBSCRIPTION IN ADVANCE WE GIVE A GOOD, RELIABLE THERMOMETER SIMPLY FOR THE ASKING, or

(2) STORIES OF A COUNTRY DOCTOR, *a most interesting book*, or

(3) ANTISEPSIS AND ANTISEPTICS, *a useful and interesting medical work.*

To old subscribers offers number two and three are open, upon the payment of one year's subscription in advance.

Other premiums, which cost you nothing, will be offered from time to time.

There is no better monthly journal published. It is published in your section and the most of its reading matter comes from men who are doing exactly the kind of work which you are doing.

By taking THE JOURNAL many of you keep in touch with former friends and fellow workers. Send in your subscriptions, get THE JOURNAL and premium, and then read THE JOURNAL.

